# Characteristics and survival of patients with metachronous or synchronous double primary malignancies: breast and thyroid cancer

**DOI:** 10.18632/oncotarget.9547

**Published:** 2016-05-20

**Authors:** Li Zhang, Yansheng Wu, Fangfang Liu, Li Fu, Zhongsheng Tong

**Affiliations:** ^1^ Department of Breast Oncology, Key Laboratory of Breast Cancer Prevention and Therapy, National Clinical Research Center for Cancer, Tianjin Medical University Cancer Institute and Hospital, Tianjin, P.R.China; ^2^ Department of Maxillofacial and Otorhinolaryngology Head and Neck Surgery, National Clinical Research Center for Cancer, Tianjin Medical University Cancer Institute and Hospital, Tianjin, P.R. China; ^3^ Department of Breast Cancer Pathology and Research Laboratory, Key Laboratory of Breast Cancer Prevention and Therapy, National Clinical Research Center for Cancer, Tianjin Medical University Cancer Institute and Hospital, Tianjin, P.R. China

**Keywords:** breast cancer, thyroid cancer, double primary malignancies, clinicopathologic characteristics, prognosis

## Abstract

**Background:**

Clinical experiences suggest that breast cancer (BC) and thyroid cancer (TC) occur metachronously or synchronously in a patient more frequently than it would by chance. This study was conducted to investigate the clinicopathological characteristics and survival of these double primary malignancies.

**Methods:**

18732 patients with first primary BC and 12877 female patients with first primary TC were performed in this retrospective case-controlled study. The control groups were matched with both age at diagnosis and time of surgery (±2 years). The clinicopathological factors, Overall survival (OS), and HRs were evaluated by SPSS.

**Results:**

There were 91(0.49%) BC patients developed metachronous second primary TC (B-T group), and 117 (0.91%) TC patients developed metachronous second primary BC (T-B group). The expression of estrogen and progesterone receptors, and the value of Ki-67, were significantly higher in the B-T group than control. The median value of thyroid globulin antibody (TGAb) and thyroid peroxidase antibody (TPOAb) were higher in T-B group than control (*p* <0.05). The duration before second primary cancer was shorter for the B-T group than the T-B group (4.09 years vs. 5.82 years, *p*<0.001). B-T group patients showed poorer survival than BC only patients (*p*=0.044).

**Conclusions:**

In general, the overall risk of the occurrence of a second primary TC or BC elevated highly in patients with BC or TC. Detailed mechanisms need to be studied to explore the association between these two cancers. Early detection and effective prevention for the first primary BC or TC patients are necessities for reducing the incidence of the second primary cancer and improving the OS.

## INTRODUCTION

Globally, breast cancer (BC) and thyroid cancer (TC) are two of the most common malignancies among women. The incidences of BC and TC are increasing in China. With the advances of cancer treatment and early detection, patients' survival has improved. However, more and more patients acquire multiple primary cancers (MPCs) because of varied reasons, such as environmental modifications, genetic predisposition, therapy, increased surveillance, or prolonged survival.

The criteria for diagnosing multiple primary tumors are as follows: 1) each tumor must present a definite picture of malignancy; 2) each tumor must be distinct; and 3) the probability that one tumor is ­­­­a metastatic lesion originating from the other must be excluded. Patients with metachronous cancer are defined as those diagnosed with a secondary cancer 6 months or more after their primary cancer diagnosis; patients with synchronous cancer are defined as those diagnosed with a secondary cancer half a year after their primary cancer diagnosis [1].

Clinical experiences suggest that BC and TC occur synchronously or metachronously in a patient more frequently than it would by chance. Some studies have reported an increased risk of TC among BC patients [2, 3], whereas others have found an increased risk of BC in TC patients [4–6]. It seems that TC and BC may have some possible associations in terms of genesis and development, such as hormonal, genetic, environmental, or therapeutic factors. It is important to know how much the risk of additional tumors increases after a primary tumor (BC or TC), as well as the differences in the clinical, pathological, and treatment characteristics among patients with and without the second primary cancer (BC or TC).

## PATIENTS AND METHODS

### Population

A retrospective case-controlled study was performed at Tianjin Medical University Cancer Institute and Hospital, Tianjin, China. The study included 18732 patients with first primary BC who underwent curative surgery and 12877 female patients with first primary TC who underwent total thyroidectomy between January 2001 and December 2010. Patients with medullary or anaplastic type of TC and those with follow-ups of less than two years were excluded. And this study conforms to the STROBE (The Strengthening of Reporting of Observational Studies in Epidemiology) statement.

During the follow up, the BC followed by metachronous second primary TC was defined as B-T group. TC followed by metachronous second primary BC was defined as T-B group. The synchronous double primary BC and TC was defined as B = T group. The control groups, all female, were matched with a ratio of 1:4 B-T and T-B groups. Matching was based on both age at diagnosis and time of surgery (±2 years). These groups were selected on a case-by-case basis from the BC or TC patients who had not been diagnosed with second primary cancer, i.e., defined as B only and T only group separately.

### Clinicopathological characteristics

We collected the clinicopathological characteristics of BC and TC, such as age at diagnosis, family history, pathologic types, tumor size, lymph nodes (LN) metastasis, distant metastasis, histological grading, estrogen receptor (ER), progesterone receptor (PR), human epidermal growth factor receptor 2 (HER2), molecular classification, Ki-67, p53, multifocality, extrathyroidal extension, thyroid stimulating hormone (TSH), thyroglobulin (TG), thyroid globulin antibody (TGAb), and thyroid peroxidase antibody (TPOAb). Immunohistochemistry was performed using the avidin-biotin-immunoperoxidase technique for ER, PR, HER2, Ki-67, and p53 in the formalin-fixed paraffin embedded representative tumor sections of each case.

**Figure 1:**
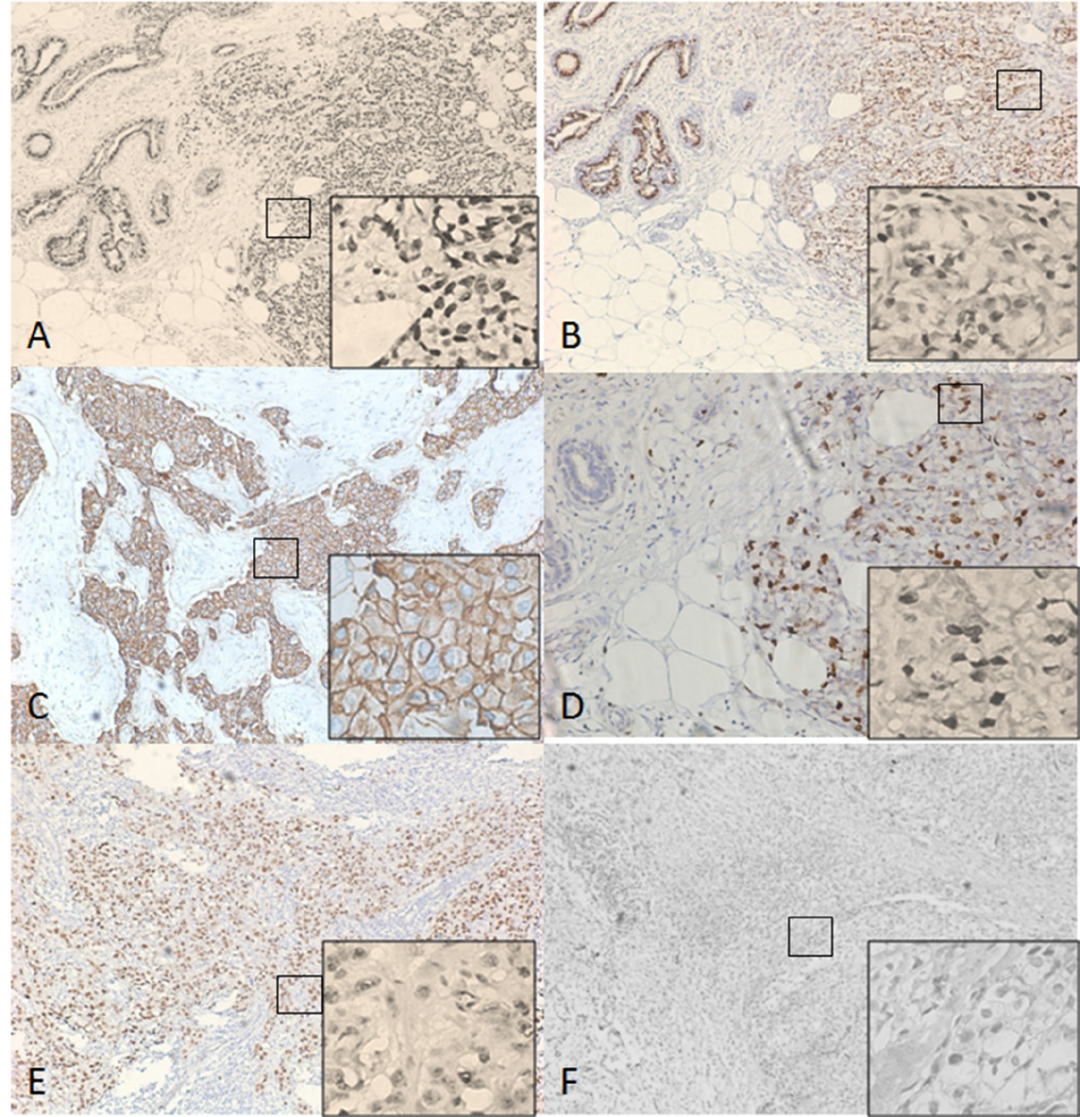
Immunohistochemistry(IHC) Images of ER, PR, Her-2, Ki67 and P53 (×100; ×400) **A.** Estrogen receptor (ER) positive; **B.** Progesterone receptor (PR) positive; **C.** Human epidermal growth factor receptor 2 (HER2) positive; **D.**, **E.** Immunoreactions of Ki-67 and p53 were presented through nuclear staining; **F**. Negative Immunoreactions.

The ER, PR, and HER2 status was determined using the criteria of the American Society of Clinical Oncology/College of American Pathologists [7, 8]. For ER and PR, nuclear staining in ≥ 1% of the tumor cells was considered positive. HER2 immunoreactivity was evaluated on a standardized scale from 0 to 3 based on the intensity of membranous staining and the proportion of tumor cells stained, wherein a strong complete membranous staining in > 10% of tumor cells (3+) was considered positive. Ki-67 and p53 immunoreactions were presented through nuclear staining (Figure 1). Molecular classification of tumor was performed using the established criteria [9, 10].

### Statistical analysis

SPSS 22.0 software was used for statistical analyses. To compare the clinicopathological characteristics of patients, chi square was used for dichotomous variables. Continuous variables were compared using the independent two-sample *t*-test. Nonparametric test was used to analyze the ranked data and continuous data not normal distributed. The standardized incidence ratio (SIR) was calculated to assess the risk of second primary malignancies by comparing the number of patients with subsequent cancer to the number of cancers that would be expected based on incidence rates for the general Chinese population [11]. Associations of different groups and the relevant tumor characteristics were explored using ANOVA and post-hoc test (LSD). Overall survival (OS) was calculated from the date of surgery. Survival curves were plotted using the Kaplan-Meier method, and group differences in survival curve were investigated by the log-rank test. A Cox proportional hazard model was used to identify variables that were independently associated with OS. All statistical tests were two-sided and a *p*-value < 0.05 was considered statistically significant.

## RESULTS

### Incidence of co-existing BC and TC

Among 18732 patients with BC, 99(0.53%) were diagnosed with synchronous double primary cancers (SDPCs) and 217(1.1%) were diagnosed with metachronous double primary cancers(MDPCs). The top five cancer types of the double primary cancers (DPCs) after BC were thyroid cancer, endometrial cancer, cervical cancer, stomach cancer and lung cancer. The incidence of thyroid cancer (0.49%) is obviously higher than other types (Table 1).

**Table 1:** The top five distribution of synchronous and metachronous double primary cancers in 18732 cases of breast cancer patients

DPC type	No.of MDPCs (%) (Total BC, *n*=18732)	No.of SDPCs (%) (Total BC, *n*=18732)
Total DPC	217(1.1)	99(0.53)
Thyroid cancer	91(0.49)	53(0.28)
Endometrial cancer	22(0.12)	12(0.06)
Cervical cancer	19(0.10)	7(0.04)
Stomach cancer	17(0.09)	7(0.04)
Lung cancer	12(0.06)	5(0.03)

Abbreviations: DPC: Double primary cancer, MDPCs: Metachronous double primary cancers, SDPCs: Synchronous double primary cancers

Among 12877 patients with TC, 83(0.64%) were diagnosed with SDPCs and 232(1.8%) were diagnosed with MDPCs. The top five cancer types of DPCs after TC were breast cancer, stomach cancer, endometrial cancer, ovarian cancer, cervical cancer. The incidence of breast cancer (0.91%) is obviously higher than other types (Table 2).

In our study, there were 91(0.49%) patients in B-T group, 117 (0.91%) patients in T-B group, and 53 cases in B = T group. During the entire follow-up period, there were 261(0.83%) patients diagnosed with co-existing BC and TC.

The estimated incidence of BC was 37.86/100,000 and TC was 10.32/100,000 in China, 2011 [11]. In patients with BC, the incidence of TC development (0.49%)was increased compared to that of the general population; the SIR for developing second primary TC was 4.75 [confidence interval (CI) 3.83-5.96]. The incidence of BC (0.91%) also increased in TC patients; the SIR for developing second primary BC was 2.40(CI 1.87-3.01).

**Table 2:** The top five distribution of synchronous and metachronous double primary cancer in 12877 cases of thyroid cancer patients

DPC type	No.of MDPCs (%) (Total TC, *n*=12877)	No.of SDPCs (%) (Total TC, *n*=12877)
Total DPC	232(1.8)	83(0.64)
Breast cancer	117(0.91)	53(0.41)
Stomach cancer	25(0.19)	11(0.09)
Endometrial cancer	21 (0.16)	4(0.03)
Ovarian cancer	15(0.11)	3(0.02)
Cervical cancer	11(0.09)	3(0.02)

Abbreviations: DPC: Double primary cancer, MDPCs: Metachronous double primary cancers, SDPCs: Synchronous double primary cancers

### Clinicopathological characteristics of BC in B-T group and B only group

The clinicopathological characteristics of BC were compared between the 91 patients in the B-T group and the 364 matched controls in the B only group (Table 3). The respective mean age and menopausal status at diagnosis of BC, family history, pathologic types, histological grading, tumor size, LN metastasis, distant metastasis, Molecular Classification, and p53 expression were similar between the two groups. The expression of both the ER and PR was significantly higher in the tumors from individuals in the B-T group compared with those from the B only group (ER+ 76.9% *vs*. 65.1%, *p* = 0.034; PR+ 74.7% *vs*. 62.1%; *p* = 0.027). The median value of Ki-67 was significantly higher (30 *vs*. 15, *p* = 0.036) in B-T group compared with B only group (Table 3).

**Table 3:** Clinicopathological Characteristics of the B-T group

Characteristics	B-T group (*n*=91)	B only group (*n*=364)	t/*X*^2^/z	*P* value
Age at first primary cancer (year, mean±s)	47.88±9.55	47.94±9.53	0.054*	0.957
Post-menopause, *n*(%)	33(36.3)	157(43.1)	1.412#	0.284
Family history, *n* (%)	7(7.7)	23(6.3)	0.223#	0.638
Pathologic types (%)			−0.195**	0.845
IDC/DCIS/other	84.6/11.5/3.9	86.2/11.3/2.5		
Maximal tumor size, *n*(%)			−0.610**	0.542
≤2cm	39(42.9)	151(41.5)		
2-5cm	38(41.8)	140(38.5)		
>5cm	14(15.4)	73(20.1)		
LN metastasis, *n*(%)	25(27.5)	109(29.9)	0.214#	0.701
Distant metastasis, *n* (%)	2(2.2)	8(2.2)	0.000#	1.000
Histological grading (%)			−0.107**	0.915
Nottingham I/ II/ III	8.8/73.6/17.6	8.2/75.3/16.5		
ER+, *n* (%)	70(76.9)	237(65.1)	4.629#	0.034
PR+, *n* (%)	68(74.7)	226(62.1)	5.085#	0.027
HER2+, *n* (%)	25(27.5)	113(31.0)	0.439#	0.610
Molecular classification, *n*(%)			−0.042**	0.967
Luminal A	55(60.4)	208(57.1)		
Luminal B	15(16.5)	89(24.5)		
HER2 positive	10(11.0)	25(6.9)		
Triple negative	11(12.1)	42(11.5)		
Ki-67(median, InterQuartile Range)	30(5~50)	15(5~40)	−2.323**	0.036
P53 (median, InterQuartile Range)	15(0~35)	10(0~30)	−0.419**	0.342

* compared using the independent two-sample *t*-test

# compared using chi square test

** compared using nonparametric test

Abbreviations: BC, breast cancer; TC, thyroid cancer; B-T group, BC followed by TC metachronously; B only group, BC patients without other second primary cancers(1:4 matched with the B-T groups); IDC, infiltrative ductal carcinoma; DCIS, ductal carcinoma *in situ*;

### Clinicopathological characteristics of TC in T-B and T only groups

The clinicopathological characteristics of TC were compared between the 117 patients in the T-B group and the 468 matched controls in the T only group (Table 4). The values of FT3, FT4, TSH, TG, TGAb, TPOAb were record preoperatively. The mean age, age at diagnosis of TC, family history, pathologic types, tumor size, lymph node (LN) metastasis, distant metastasis, the proportion of multifocality, extrathyroidal extension, FT3, and FT4 were similar between the two groups. The median value of TGAb and TPOAb were higher in T-B group compared with that in T only group (TGAb 5.25 *vs*. 2.65, *p* = 0.008; TPOAb 7.69 *vs*. 3.54, *p* = 0.022) (Table 4).

**Table 4:** Clinicopathological Characteristics of the T-B group

Characteristics	T-B group (*n*=117)	T only group (*n*=468)	t/*X*^2^/z	*P* value
Age at first primary cancer (year, mean ± SD)	46.38±9.66	46.70±9.61	0.313*	0.755
Post-menopause, *n* (%)	52(44.4)	214(45.7)	0.062#	0.836
Family history, *n* (%)	13 (11.1)	39(8.3)	0.892#	0.364
Histologic type (%)			0.266#	0.747
PTC/ FTC	89.7/10.3	88.0/12.0		
Maximal tumor size, *n* (%)			−1.315**	0.188
≤2cm	51(43.6)	177(37.8)		
2-4cm	42(35.9)	172(36.8)		
>4cm	24(17.9)	119(15.2)		
LN metastasis, *n*(%)	37(31.6)	174(37.3)	1.287#	0.283
Distant metastasis, *n* (%)	1(0.9)	7(1.5)	0.285#	1.000
Multifocality, *n* (%)	30(25.6)	110(23.5)	0.235#	0.629
Extrathyroidal extension, *n* (%)	34(29.1)	121(25.9)	0.494#	0.484
FT3 (pmol/L, median, InterQuartile Range)	7.23(5.24~9.66)	7.67(5.12~9.92)	−1.634**	0.102
FT4 (pmol/L, median, InterQuartile Range)	10.12(8.21~12.13)	10.37(7.91~12.34)	−1.516**	0.130
TSH (mIU/L, median, InterQuartile Range)	2.67(1.96~3.42)	2.88(2.12~3.45)	−1.645**	0.101
TG (μg/L, median, InterQuartile Range)	59.23(29.89~70.25)	57.11(28.21~69.35)	−1.034**	0.218
TGAb (IU/ml, median, InterQuartile Range)	5.25 (2.68~8.38)	2.65 (1.95~5.34)	−2.672**	0.008
TPOAb (IU/ml, median, InterQuartile Range)	7.69(3.52~10.24)	3.54(1.12~5.94)	−2.124**	0.022

* compared using the independent two-sample t-test

# compared using chi square test

** compared using nonparametric test

Abbreviations: BC, breast cancer; TC, thyroid cancer; T-B group, TC followed by BC metachronously; T only, TC patients without other second primary cancers(1:4 matched with the T-B groups); PTC, papillary thyroid carcinoma; FTC, follicular thyroid carcinoma.

### Difference of characteristics among co-existing BC and TC

**Figure 2:**
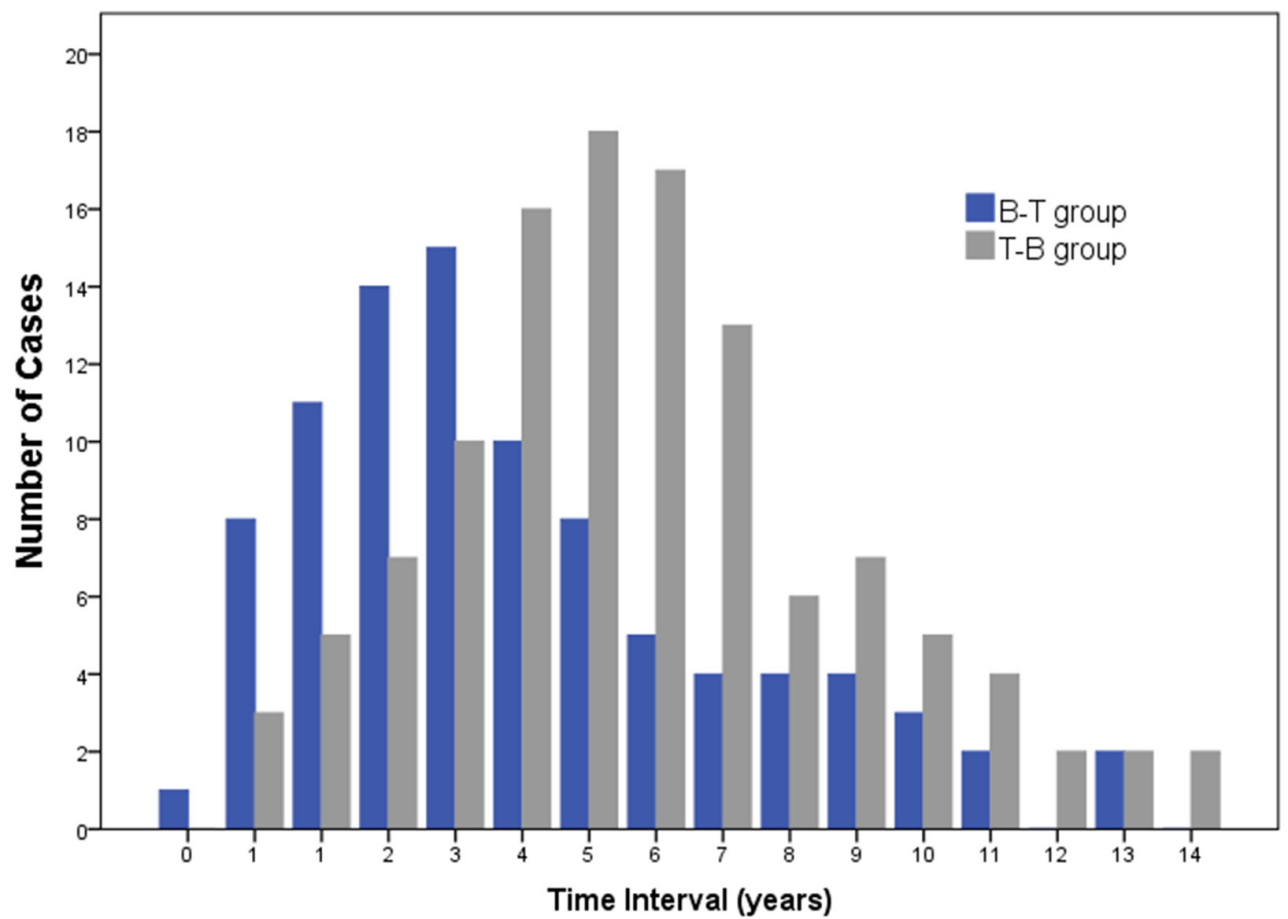
Time interval between breast cancer and thyroid cancer The mean interval time before second primary cancer is shorter for the B-T group than the T-B group (4.09 years *vs*. 5.82 years, *p* < 0.001)

Among the 18732 BC and 12877 TC patients, 261 were diagnosed with co-existing BC and TC (B-T: 34.9%, T-B: 44.8%, B = T: 20.3%) after the follow-up period (Table 5). The age at first primary cancer is significantly different among the three groups (B = T: 43.66 *vs*. T-B: 47.88, T-B: 46.38). Analyzed by post-hoc test, patients in B = T group were younger than B-T groups (*p* = 0.012). The mean interval time before second primary cancer is shorter for the B-T group than the T-B group (4.09 years *vs*. 5.82 years, *p* < 0.001) (Figure 2). The BC and TC characteristics, such as ER, PR, Her-2, Ki-67, P53, FT3, FT4, TSH, TG, TGAb, and TPOAb, were similar among B-T, T-B, and B = T groups (Table 5).

**Table 5:** Difference of characteristics among co-existing BC and TC

Characteristics	Co-existing BC and TC (n=261)			*P* value
	B-T *n*=91 (34.9%)	T-B *n*=117 (44.8%)	B=T *n*=53 (20.3%)	
Age at first primary cancer (year, mean ± SD)	47.88±9.55*	46.38±9.66	43.66±10.03*	0.044
Duration before second primary cancer (years, mean± SD)	4.09±3.13	5.82±3.04	—	<0.001
BC characteristics				
ER+, *n*(%)	70(76.9)	95(81.2)	40(75.5)	0.629
PR+, *n* (%)	68(74.7)	93(79.5)	38(71.7)	0.497
HER2 (%)	25(27.5)	31(26.5)	19(35.8)	0.434
Ki-67	30(5~50)	30(5~50)	25(5~45)	0.234
P53	10(0~35)	15(0~40)	10(0~35)	0.901
TC characteristics				
FT3 (pmol/L, median InterQuartile Range)	7.13(5.44~9.64)	7.23(5.24~9.66)	7.25(5.34~9.56)	0.643
FT4 (pmol/L, median InterQuartile Range)	10.10(8.54~12.45)	10.12(8.21~12.13)	10.64(8.41~13.03)	0.223
TSH (mIU/L, median InterQuartile Range)	2.34(1.33~3.34)	2.67(1.96~3.42)	2.87(1.90~3.32)	0.742
TG (μg/L, median InterQuartile Range)	60.77(29.78~71.24)	59.23(29.89~70.25)	59.44(26.89~70.66)	0.256
TGAb (IU/ml, median InterQuartile Range)	5.87 (1.92~8.33)	5.25 (1.98~8.38)	5.23 (2.18~8.88)	0.811
TPOAb (IU/ml, median InterQuartile Range)	7.33(3.23~10.22)	7.69(3.52~10.24)	7.67(3.77~10.74)	0.245

* analyzed by post-hoc test, *p* = 0.012.

Abbreviations: BC, breast cancer; TC, thyroid cancer; B-T, BC followed by TC metachronously; B=T, BC and TC occurred synchronously ; T-B, TC followed by BC metachronously; Co-existing BC and TC, total cases of B-T group, B=T group and T-B group.

### Survival analysis

**Figure 3:**
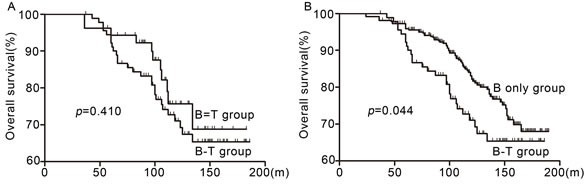
Comparison of OS between B-T group, B = T and B only group **A.** B-T group showed similar survival with B = T group (*p* = 0.410). **B.** B-T group showed poorer survival than the B only group (*p* = 0.043).

The survival curves of B-T and B = T, B-T and B only groups are shown in Figure 3. We did not report the survival data of T-B group because we will obtain a worse OS compared to that of T only group with no controversy.

The median OS of B-T group, B = T and B only group were 110, 112 and 118 months separately. Five year OS rates were 88.8%, 89.2% and 96.7%, whereas 10 year OS rates were 69.7%, 73.1% and 78.5%. B-T group patients showed similar survival with B = T group (*p* = 0.410) (Figure 3A), but showed poorer survival than the B only group (*p* = 0.044) (Figure 3B). The survival curve of B-T, B = T and B only group showed obvious decline at about 60, 80 and 100 months separately after diagnosis.

In multivariate Cox regression analysis, after adjusting the factors of maximal tumor size, LN metastasis, and distant metastasis at diagnosis of breast cancer, patients in B-T group showed a significant increase in the risk of death compared with B only group (HR 2.261, 95% CI 1.378-3.710, *p* = 0.001) (Table 6).

**Table 6:** Multivariate Cox regression prognostic analysis of OS

Factors	Hazard ratio	95% confidence interval	*p* value
Maximal tumor size			
≤2cm	Ref.		
2-4cm	0.445	0.260-0.760	0.003
>4cm	0.446	0.253-0.787	0.005
LN metastasis			
Negative	Ref.		
Positive	0.096	0.057-0.160	<0.001
Distant metastasis			
Negative	Ref.		
Positive	0.309	0.146-0.651	0.002
Groups			
B-T	Ref.		
B only	2.261	1.378-3.710	0.001

Abbreviations: LN, lymph nodes

## DISCUSSION

In our study, 0.49% of the BC and 0.91% of the TC patients were diagnosed with metachronous TC and BC, respectively. In patients with BC, the incidence of TC development increased compared with the general population. The SIR for developing second primary TC was 4.75. The incidence of BC also increased in TC patients with a SIR for developing second primary BC of 2.40. These revealed that patients with TC or BC have a higher risk of developing second primary BC or TC. The SIR value was calculated according to the estimated incidence of BC (37.86/100,000) and TC (10.32/ 100,000) in China in 2011 [11]. The SIR value of TC was higher compared with the value obtained in previous studies (from 1.2 to 4.6) [12–15]. The increased incidence of TC followed by the occurrence of BC might be attributed to the incidental detection of TC during follow-up of BC or to the frequent health check-ups following BC. Besides, obvious differences existed in incident area or time. Across U.S. counties, incidence of TC ranged widely, from 0 to 29.7 per 100,000 [16], whereas the incidence was 10.32 per 100,000 in China 2011 [11].

In the B-T group, the expression of ER and PR was significantly higher, and the median Ki-67 labeling was higher than that of B only group. In accordance with our result, some previous studies have also suggested for the role of hormone receptors in the molecular pathogenesis of TC. Sex steroid receptors were found in human thyroid tissue, several TC cell lines, and ER levels were significantly higher in TC compared with normal thyroid tissue [17–19], which have shown the possibility that ER or PR signaling might represent common etiological factors in the development of TC and BC. Other studies have shown that estrogen could up-regulate the expression of cell cycle-related genes and proto-oncogene in thyroid cells, which were likely to contribute to the development of TC [20]. Proliferative activity of tumor tissue is commonly measured by Ki67, which is a well-established prognostic and a predictive marker [21]. Higher expression of Ki67 could be detected in breast-ovarian cancer syndrome (BOCS) [22]. In our study, the incidence of TC was increased in BC patients with higher Ki-67.

Compared with T-B and T only group, we found that the mean value of TGAb and TPOAb were higher in T-B group. Previous studies showed that serum levels of TPOAb and TGAb were significantly higher in patients with BC than in healthy people [23, 24]. Another study showed a poor prognosis in BC with higher TPOAb [25]. However, other studies found that TPOAb positivity was associated with a lower incidence of metastasis in BC patients [26]. Our study indicated that not only the healthy population but also the TC patients should pay more attention to the increase of TGAb and TPOAb. Regular breast examination is important for TC patients.

Among the 18732 BC patients and the 12877 TC patients, 261 were diagnosed with co-existing BC and TC over the follow-up period. The mean interval time before second primary is shorter for B-T group than T-B group (4.09 years *vs*. 5.82 years, *p* < 0.001). Close monitoring for the detection of TC development might be necessary for patients with BC, especially 4-5 years after primary diagnosis. Moreover, regular breast examination should be emphasized among TC patients, especially 5-6 years after primary diagnosis. The age at first primary cancer is significantly different among the three groups. Patients in B = T group were younger than B-T groups. The mean age was 44 years old. Other studies showed that the mean duration before the occurrence of second primary cancer was different for different types of cancers and areas(3.1-8.5 years) [27–30]. In our study, results indicated that co-existing BC and TC patients, whether synchronous or metachronous, possessed similar characteristics of ER, PR, Her-2, Ki-67, P53, FT3, FT4, TSH, TG, TGAb, and TPOAb values.

There are limited data on the effect of second primary cancer of thyroid on the survival of BC patients. In the M.D. Anderson Cancer Center, a study on 4198 patients subjected to breast conservation therapy showed that the patients with MPCs showed worse OS than those without MPCs [31]. Similarly, patients with MPCs in another study demonstrated worse DFS and OS [32]. In our study, more deaths due to cancer occurred in the B-T group than in the B only group. Although thyroid cancer usually has better prognosis, it increased the death rate of patients with BC as a second primary cancer. BC patients with metachronous and synchronous double primary TC showed similar survival. We did not report the data of survival on T-B group because we will obviously obtain OS statistics that is worse than T only group with no controversy.

In conclusion, this study has identified that that the overall risk of having a second primary TC or BC is increased in patients with BC or TC. BC patients with higher expression of ER, PR, or Ki-67 should pay more attention to the development of TC, especially 4-5 years after breast surgery. TC patients with higher TGAb and TPOAb have a higher risk of obtaining a second primary BC. Thus, regular breast examination should be emphasized among these patients, especially 5-6 years after thyroid surgery. Patients with co-existing BC and TC usually exhibited worse survival than those with only BC or TC. Therefore, further efforts are needed to explore the mechanism, develop early detection, and administer effective prevention for patients with second primary cancers.
